# Coupling Effects in Multistage Laser Wake-field Acceleration of Electrons

**DOI:** 10.1038/s41598-019-56654-x

**Published:** 2019-12-27

**Authors:** Zhan Jin, Hirotaka Nakamura, Naveen Pathak, Yasuo Sakai, Alexei Zhidkov, Keiichi Sueda, Ryosuke Kodama, Tomonao Hosokai

**Affiliations:** 1 Institute of Scientific and Industrial Research, Osaka University, 8-1 Mihogaoka, Ibaraki, Osaka, 567-0047 Japan; 2 Laser Acceleration Development Team, Innovative Light Sources Division, RIKEN SPring-8 Center, 1-1-1, Kouto, Sayo-cho, Sayo-gun, Hyogo, 679-5148 Japan; 3 Graduate School of Engineering, Osaka University, 2-1 Yamada-oka, Suita, Osaka, 565-0871 Japan

**Keywords:** Laser-produced plasmas, Plasma-based accelerators

## Abstract

Staging laser wake-field acceleration is considered to be a necessary technique for developing full-optical jitter-free high energy electron accelerators. Splitting of the acceleration length into several technical parts and with independent laser drivers allows not only the generation of stable, reproducible acceleration fields but also overcoming the dephasing length while maintaining an overall high acceleration gradient and a compact footprint. Temporal and spatial coupling of pre-accelerated electron bunches for their injection in the acceleration phase of a successive laser pulse wake field is the key part of the staging laser-driven acceleration. Here, characterization of the coupling is performed with a dense, stable, narrow energy band of <3% and energy-selectable electron beams with a charge of ~1.6 pC and energy of ~10 MeV generated from a laser plasma cathode. Cumulative focusing of electron bunches in a low-density preplasma, exhibiting the Budker–Bennett effect, is shown to result in the efficient injection of electrons, even with a long distance between the injector and the booster in the laser pulse wake. The measured characteristics of electron beams modified by the booster wake field agree well with those obtained by multidimensional particle-in-cell simulations.

## Introduction

The laser wake-field acceleration (LWFA) of electrons is one of the rapidly developing scientific fields of the last decade^1–17^. This technique, providing potentially jitter-free sources of radiation and electrons, has already demonstrated an electron acceleration of ~8 GeV^13,14^ in a single stage with laser pulse energy less than 100 J. Recently, interest in schemes with external injection^18^ has rapidly grown, with staging schemes being developed. Similar to conventional radio frequency acceleration schemes, the staging schemes in plasma (an injector, a buster, etc.) seem to be more practical, providing better scalability, stability and reproducibility of the acceleration process^19,20^. Such schemes exploiting spatially separated injectors and boosters^20^ have clear technical advantages in contrast to ‘overlapped plasma’ schemes^19^. However, beam delivery or coupling in this case becomes a critical issue.

Coupling in the spaced-apart staging schemes for LWFA is apparently a key problem. Coupling problems originate from a possible mismatch in the sizes of the injected electron bunches and the sizes of the laser wake fields. The transverse size of a laser wake field is limited by the value of *a*_0_ (the normalized vector potential of the laser field, *a*_0_ = *eE*_L_/*mcω*, where *E*_L_ is the laser electric field strength and *ω* is the laser pulse frequency^21)^, which is necessary for efficient acceleration and implies an upper limit on the value of the laser pulse waist, *w*_0_. On the other hand, the longitudinal size of the acceleration field is determined by the plasma electron density: the smaller the electron density is, the longer the wave of the laser pulse wake. However, laser pulse guiding in plasma becomes possible with an electron density *N*_*e*_ > *1.7* *×* 10^10^ *N*_*cr*_*[cm*^*−3*^*]/P[W]*, where *N*_*cr*_ is the critical density for the laser pulse frequency and *P* is the total power of the laser pulse. Moreover, an electron density that is too low results in a weaker acceleration field strength *E* = *a*_0_ *λ/λ*_*p*_, where *λ* is the laser wavelength and *λ*_*p*_ = *λ(N*_*cr*_*/N*_*e*_)^*1/2*^ is the plasma wavelength. The sizes of electron bunches coming out of the injectors are determined by (i) the geometrical emittance and (ii) the energy spread *Δγ* (*γ* is the relativistic factor, *γ* = [*1* + *(p/mc)*^2^]). If a distance between an injector and a booster is *L*, the bunch length at the entrance point will be equal to *l* = *LΔγ/γ*_0_^3^, where *γ*_0_ is the mean energy of electrons in the bunch. [We assume that *Δγ* << *γ*_0_ and that *l* exceeds the initial bunch length]. In the case of *l* > *λ*_*p*,_ an essential portion of electrons cannot be injected into the acceleration phase of the laser wake field.

It is also clear that if the bunch transverse size exceeds the laser pulse diameter in the focus spot, an essential part of the injected electron cannot be accelerated. For example, the injection efficiency of an electron bunch with a diameter of ~1 mm into a wake generated by a laser pulse with a focus spot of ~20 *μ*m is only 0.04% or almost zero. However, this coupling cannot be simply estimated using only the geometrical emittance of bunches. Fortunately, the actual efficiency may be quite high due to the Budker–Bennett effect^22,23^ in plasma. The injection of high-energy electron beams in plasma results in evacuation of some plasma electrons from the beam axis owing to the beam longitudinal electric field. This process is the beginning of the formation of a beam wake field^24^. Electrons in beams are heavier (by a factor of *γ*_0_) than plasma electrons and cannot be evacuated. Therefore, beam electrons propagate over a positively charged part of the plasma. To estimate the effect of this plasma on beam electrons, we use the Poisson equation in the reference frame moving with the beam electrons to exclude the magnetic field. One can easily find that the electric field strength obeys the following equation:

1 $$\frac{1}{r}\frac{\partial }{\partial r}(r{E}_{tr})=-\,4\pi e[{N}_{B}/{\gamma }_{0}-{\gamma }_{0}({N}_{i}-{N}_{e})],$$

where *E*_*tr*_ is the electric field strength in the transverse direction, *N*_*B*_ is the beam density, and *N*_*i*_ and *N*_*e*_ are the ion and electron density in plasma, respectively. If the value in the square brackets is positive, the beam electrons will move towards the beam axis or will be focused. The difference in the density should be (*N*_*i*_* − N*_*e*_) > *N*_*B*_/*γ*_0_^2^. For a spherical beam with a 10 *μ*m diameter and a total charge of ~10 pC, the beam density is *N*_*B*_~6 × 10^16^ cm^−3^. For *γ*_0_ = 20, *ΔN*~10^14^ cm^−3^. This condition does not require dense plasma, and beam focusing may occur even in a very-low-density plasma part in front of a plasma target.

**Figure 1:**
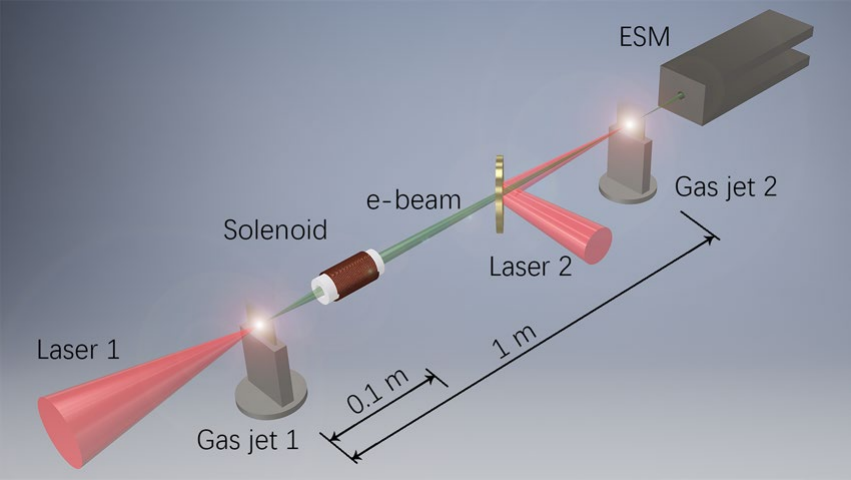
Experimental setup. Laser beam 1 was focused on a He gas jet producing injector electrons. A solenoid collected and focused the electrons with the desired energy onto the booster plasma wake-field placed 1 meter away. An electron spectrometer was installed just after the second gas jet.

In the present work, we investigate these temporal and spatial coupling effects in a booster irradiated by 450 mJ laser pulses using well-determined electron bunches generated from a laser-plasma cathode^25^ with a charge of ~1.6 pC and an energy of ~10 MeV with an energy spread *ΔE* < 3% (Fig. 1). To understand the details of the effects, we also perform multidimensional particle-in-cell simulations for both temporal coupling and electron focusing in various plasmas.

## Results

**Figure 2:**
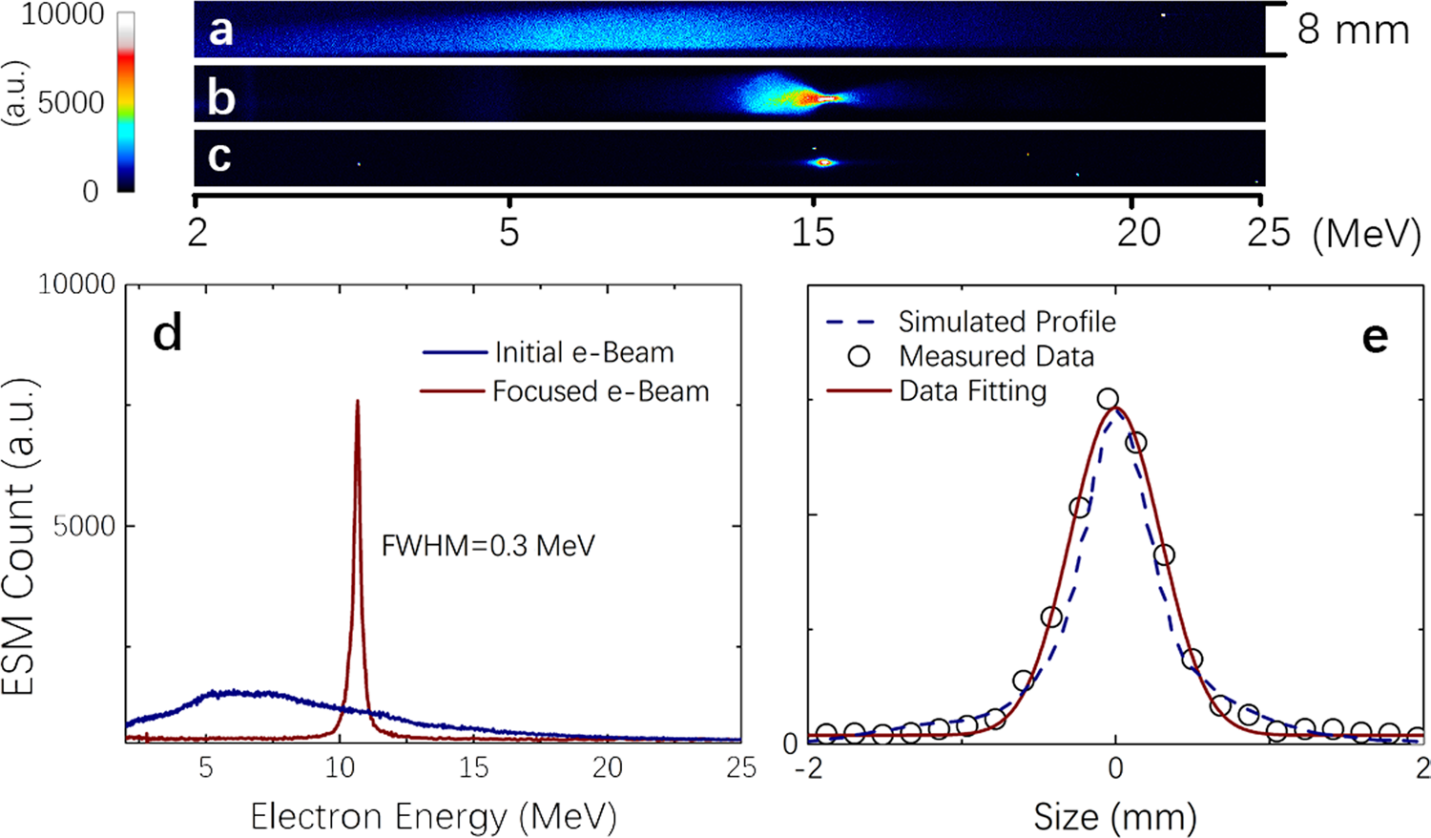
Characteristics of electron beams from the cathode: (**a**) Initial injector electron beam’s spectrum. (**b**) Spectrum with 0.8 kV applied to the solenoid. (**c**,**d**) Spectrum with the same voltage and an additional 500 *μ*m diameter, 5 mm thick molybdenum aperture at the electron focus spot. (**e**) The spatial profile of the electron focus. Electrons with a beam energy of ~10 MeV can be focused with an energy spread of approximately 3%. The focus spot size of the electrons was measured to be <0.7 mm FWHM with a total charge of ~1.6 pC. The dashed line gives the GPT simulation result.

First, we characterized the injection electron bunches in a manner similar to that described in ref. ^25^. Figure 2a–c gives the spectral image of the injector electron beam on the ESM measured without the function of a solenoid, with 0.8 kV applied to the solenoid, and with the same voltage with an additional 500 *μ*m diameter, 5 mm thick molybdenum aperture at the electron focus spot, respectively. Due to the short *f*-number parabolic mirror we used for the cathode, electron beams with the thermal-like spectrum were observed, with their maximal energy up to 25 MeV, as shown in Fig. 2d. The total charge number of electrons >1 MeV was estimated to be ~500 pC. This setup allows us to collect the specified energy of electrons at a predetermined focal point by tuning only the solenoid voltage. With an applied voltage equal to 0.8 kV, accelerated electrons with an energy of ~10 MeV were selected and focused with an energy spread of approximately 3%. The focus spot size of the electrons was measured to be <0.7 mm FWHM with a total charge of ~1.6 pC^26–28^, as shown in Fig. 2d,e. The efficiency of the energy selection from the initial cathode by the solenoid is approximately 0.3%. Particle tracking simulations (GPT from Pulsar Physics) were done with the parameters similar with our injector. The simulated beam profile (dashed like in Fig. 2e) agrees well with the measurement result.

**Figure 3:**
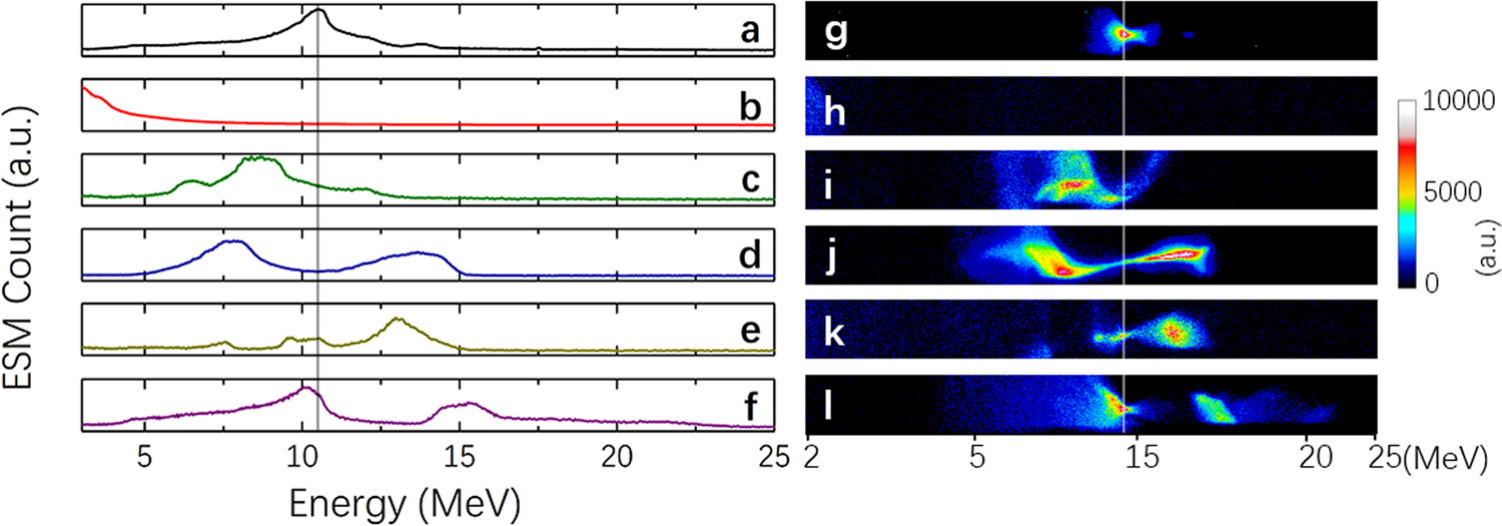
Output spectrum of staging acceleration. (**a**) Electron spectrum from the injector laser, focused by the solenoid. (**b**) Electron generated by the booster laser pulse only. (**c**–**f**) Electron spectrum with both the injector and booster. (**g**-**l**) are the raw images taken by the ESM.

To separate the spectra modified upon the interaction of the injected electron beams with the booster plasma, we first characterized the electron spectra without coupling. Figure 3a,b show the electron spectra measured for the cathode (injector) only and for the booster (dark current, without a cathode beam) only, respectively. One can see that the dark current in this experiment was negligibly small. This result was due to the rather low intensity of the second laser beam. An efficient electron self-injection in the booster plasma was absent, and no high-energy electron beam was observed. It was also reached upon careful tuning of the gas jet position.

Modulated spectra of accelerated electrons after passing the booster are shown in Fig. 3c–f, with their dependence on the delay time presented. The zero time equals the time when both the injector electron beam and booster laser beam were delivered to the booster synchronously. Clear deceleration and/or acceleration of the beam electrons were observed to depend on the time delay, exhibiting the coupling of the injection electron beams with the laser wake field in the booster. Visible instability of the measured spectra mainly came from the temporal jitter (small but notable in the present setup) and the vibration of the laser focus pointing. Note that the diameter of the injector beam focused only by the solenoid is hundreds of micrometers, which is much larger than the booster wake-field transverse size. However, the results show that most of the electrons can be modulated by the booster laser wake field, which indicates that there is possibly a self-focusing by the beam-induced plasma wake-field when the injector electrons propagate in the low-density-gas region before the main jet.

The spectrum behavior strongly depends on the geometrical parameters of the injected beam at the position of the booster, *L*~1 m. We measured the transverse size of the injected beams as 0.7 mm without the booster plasma. This large diameter is a result of the geometrical emittance of the cathode beams. The beam length *l* = *LΔγ/γ*_0_^3^ should be (for *Δγ/γ*_0_~2–3% and *γ*_0_ = 20) equal to 70–100 *μ*m. The spot size of the second laser beam is approximately *D*~20 *μ*m, and the length of the laser wake field is approximately *λ*_*p*_~10 *μ*m for the parameters of the experiment. It seemed difficult to observe an essential coupling for these circumstances. However, the coupling was observed. Moreover, the number of counts for accelerated electrons with modified energy after passing through the booster varied from 10% to 90% relative to the initial value. These values exceed the expected values by orders of magnitude! We attribute this to the effect of Budker–Bennett^22,23^. According to the measurement result^29^, the gas jets used in the experiment have a long, several mm front part with a relatively low gas density *N*~10^17^ cm^−3^. In the booster, this part of the gas flux becomes plasma after the second laser beam passes through due to optical field ionization.

**Figure 4:**
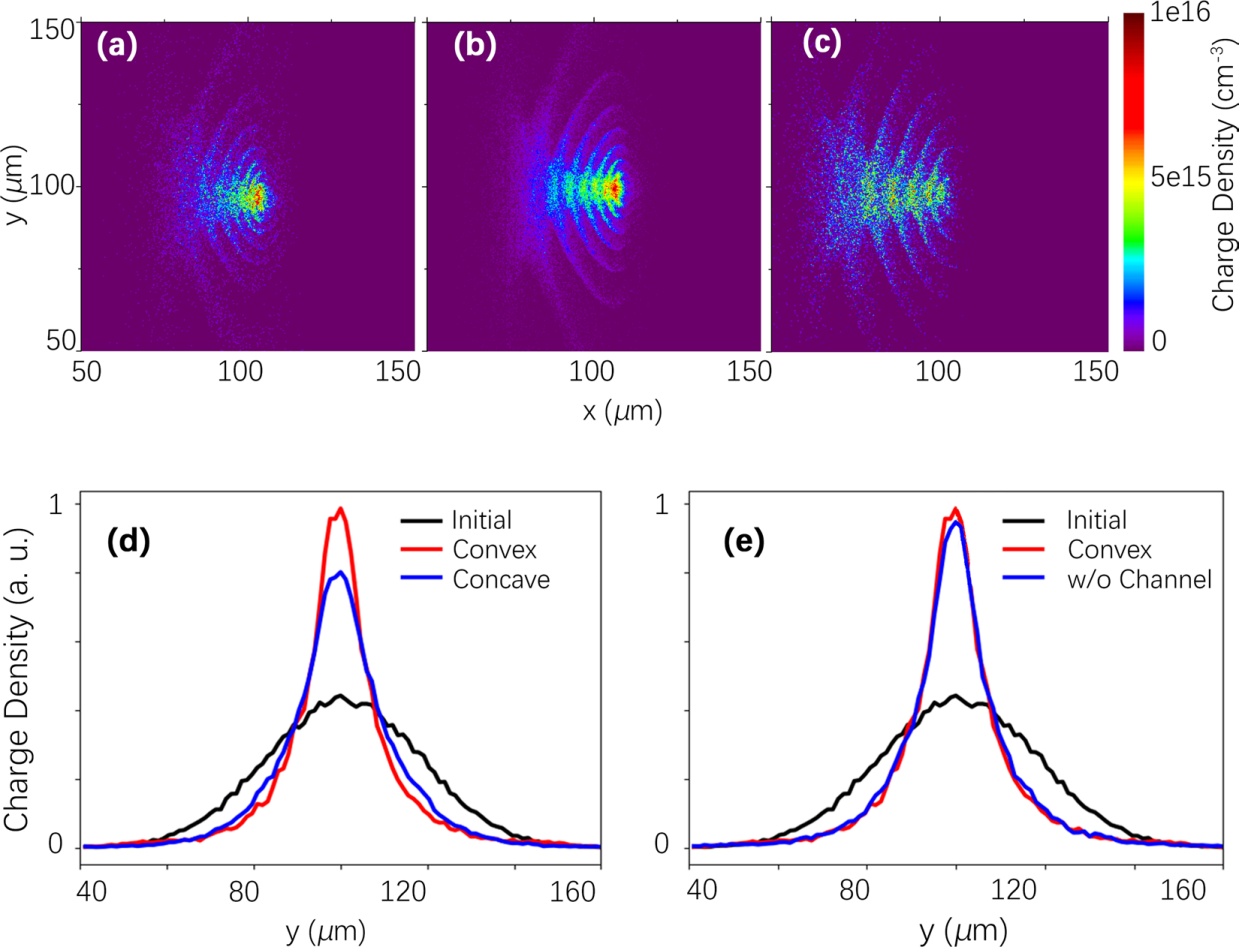
Self-focusing of an injector electron beam (Budker–Bennet effect) in low density plasma, *N*_e_ = 3 × 10^17^ cm^−3^. (**a**–**c**) give spatial distributions of the bunch electrons after 30 ps of propagation: (**a**) electron bunch, *E* = 10 MeV, propagates in a concave plasma channel with *D* = 100 *μ*m and a depth of 0.5; (**b**) electron bunch propagates in uniform plasma; and (**c**) electron bunch propagates in a convex channel with a height of 0.5. (**d**, **e**) Transverse distribution of the electron bunch after 30 ps of propagation in plasma with different shapes.

To verify this effect, we performed multidimensional particle-in-cell simulations for electron beams propagating in the low-density plasma of *N*_*e*_ = 3 × 10^17^ cm^−3^. The beam diameter was 50 *μ*m, the electron energy was *E* = 10 MeV, the energy spread was *ΔE/E* = 3%, the total charge was 10 pC, and the geometrical emittance was 10^−2^ rad. We considered three plasma targets: uniform plasma and convex and concave density plasma channels. The ratio of the minimal electron density to the maximal in the case of channels was equal to 0.7. In Fig. 4, the longitudinal and transverse beam profiles are given after 30 ps of propagation in the plasma. One can see an essential focus of the electron bunches inside all kinds of plasma targets. The weakest focusing is observed for a concave plasma channel, while the strongest focusing is observed for a convex plasma channel, as shown in Fig. 4d,f. This observation reflects a simple fact: the evacuation of plasma electrons is easier for negative density gradients. This proves that long preplasma in front of the booster can focus much more strongly the injected electron beams to a diameter sufficient for the efficient coupling of these electrons with the booster wake field. We anticipate that this process may become critical in developing beam lines for dense, high-charge electron beams generated by laser wake fields.

However, the beam length cannot be shortened by the plasma, and it remains for 10 MeV-electron bunches with a few percentage energy spread longer than the length of the wake wave. Therefore, accelerated electrons were injected in the different phases of the laser wake field in the booster. The final energy distribution of the electron should have accelerated, decelerated, and intact parts. To reveal the process of interaction of a well-determined electron beam with the laser wake field, we perform a multidimensional particle-in-cell simulation with extraction of the beam electrons from plasma electrons in postprocessing.

**Figure 5:**
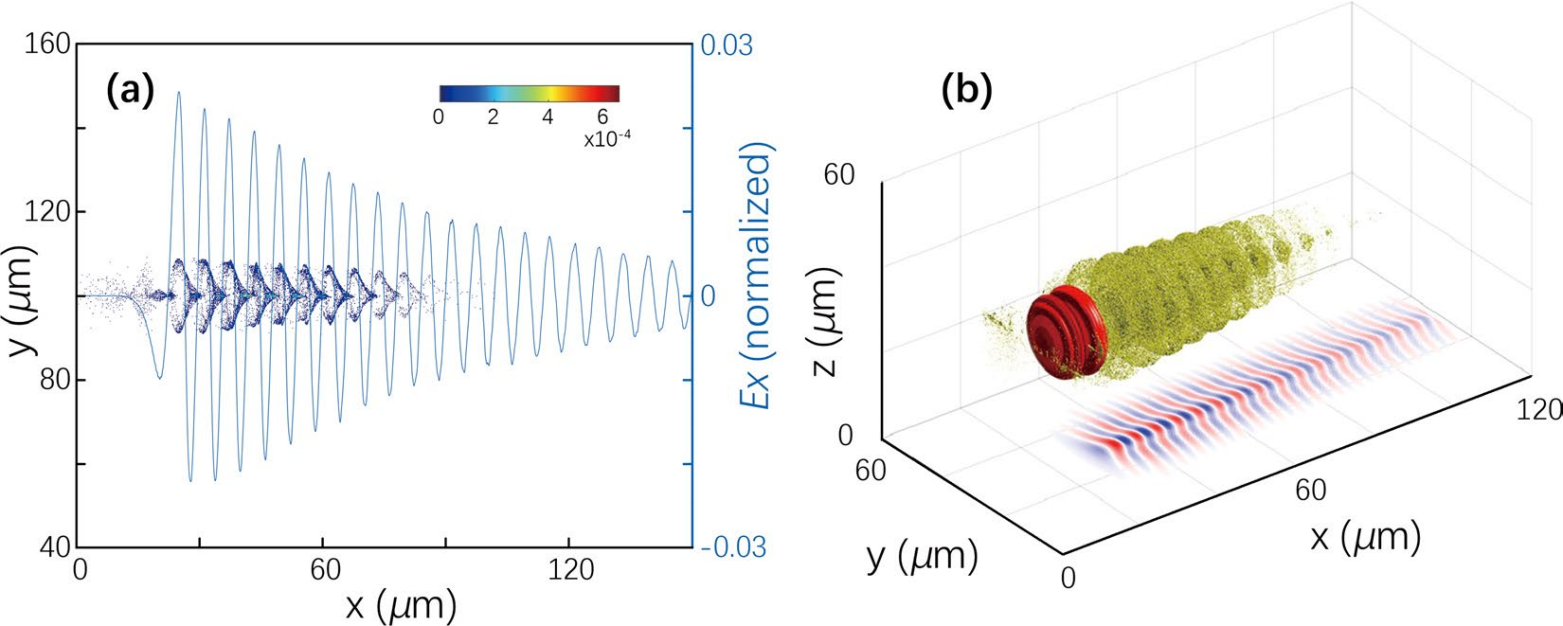
Spatial distribution of the electron bunch (*E* = 10 MeV, bunch length is 70 *μ*m) modulated by the booster laser wake field and the wake-field strength in a uniform plasma; (**a**) 1D projection and (**b**) 3D projection for the bunch density, with a 2D projection for the wake field.

The typical spatial distributions of accelerated electrons along with the electric field of the laser wake, produced by a laser pulse with a duration of 30 fs and *a*_0_~2, are given in Fig. 5a (1D projection) and in Fig. 5b (3D plot) for an electron bunch with *E* = 10 MeV and a length of 70 μm, which essentially increase the plasma wavelength. One can see a very strong modulation of the electrons in the wake field. As expected, the length of the bunch exceeds the length of the wake wave. Along with the classic theory of oscillations, most electrons are concentrated near the zero-field points. Therefore, it is difficult to reach an efficient acceleration or deceleration under the present conditions. A change in the time delay cannot help much since the beam length far exceeds the wake wavelength.

**Figure 6:**
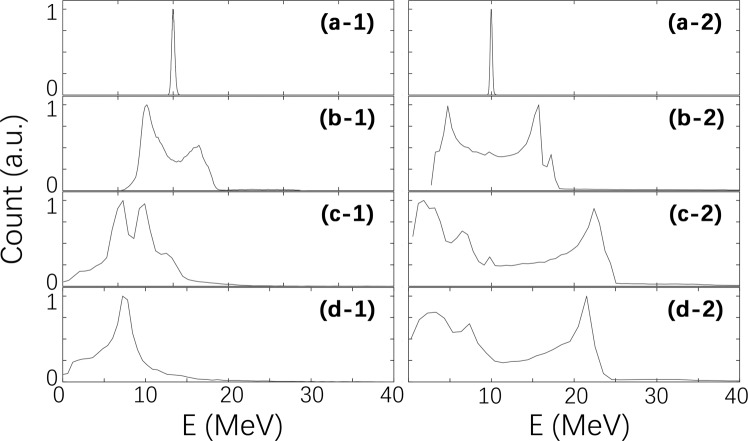
Evolution of electron beam energies during the interaction with the plasma waves in the second (booster) stage at different plasma densities. (**a**-**1**) to (**d**-**1**) shows the electron energy evolution at a plasma density of 3 × 10^19^ cm^−3^, and (**a**-**2**) to (**d**-**2**) show the electron energy evolution at a plasma density of 3 × 10^18^ cm^−3^ in the booster stage. (**a**-**1**, **a**-**2**), (**b**-**1**, **b**-**2**), (**c**-**1**, **c**-**2**), and (**d**-**1**, **d**-**2**) show the energy of the electron beam at 0.1 ps, 1.5 ps, 3.0 ps, and 4.5 ps, respectively.

The dynamics of the electron energy distribution is given in Fig. 6 up to 4.3 ps. This figure confirms that only a small number of electrons are further accelerated and, correspondingly, a small number of electrons are decelerated. Most electrons have nearly the same energy as before the interaction. This observation is in agreement with the experimental results shown in Fig. 3 and proves the strong coupling of the injected electron with the booster. We present the results of the calculation for two density values. Both *N*_*e*_ = 3 × 10^19^ cm^−3^ and *N*_*e*_ = 3 × 10^18^ cm^−3^ give similar results for the evolution of energy distributions of 10 MeV electron bunches with a 3% energy spread. However, we note that the lower density distribution is closer to the measured distribution.

## Discussion

In conclusion, we have demonstrated the strong coupling of electrons accelerated with the laser pulse from a gas jet, with wave breaking electron self-injection and the conventional beam energy slice techniques^25^, and the booster produced in the second gas jet by another laser pulse temporally and spatially synchronized with the first laser beam. First, the electron beams with an energy of 10 MeV and a charge of ~1.6 pC were focused and transported 1.4 m downstream with an energy spread of ~3% and a spatial size of less than 800 *μ*m in the FWHM, respectively. These beams were injected in the wake field generated in the second gas jet by another synchronized laser beam.

The injected efficiency was measured to be unexpectedly high, from 10% to 90% of the electron charge after the energy selection despite the low focus diameter of the second laser beam, i.e., *D*~10 *μ*m. This high efficiency is the result of the Budker–Bennet effect of electron self-focusing in rather low-density preplasma. This was proved by multidimensional particle-in-cell simulations with various plasmas. It has been shown that this effect may be used for the organization of transport lines for laser-driven dense electron beams.

The longitudinal spread of the electron bunch at the booster position was too long, ~80–100 *μ*m, to provide efficient electron acceleration in the wake wave with a size of *λ*_*p*_~10 *μ*m. The maximal energy of electrons reached ~30 MeV with a maximal gain of ~3 times. The results of multidimensional particle-in-cell simulations agree well with the observations and explain the dynamics of the interaction of the injected electrons with the laser wake field for the present experimental setup. In addition, a long injector electron bunch length exceeding the booster plasma wavelength allows for the wake-probing of all phases, including acceleration/deceleration and focusing/defocusing in a single shot.

## Methods

### Laser system

The experiment was carried out with the P-cube 80-TW Ti:Sapphire laser system (Amplitude Technologies) at Osaka University based on a chirped pulse amplification (CPA) technique^30^. The laser system can deliver 2 synchronized CPA laser beams from the same oscillator. The first laser beam (shown as Laser 1 in Fig. 1) serves as the plasma cathode. The pulse energy on a target was *E*_L1_ = 500 mJ at its maximum, and the pulse duration was *τ* = 30 fs. The booster laser beam 2 had *E*_L2_ = 350 mJ and a 30 fs duration. The central wavelength of the laser pulse was *λ* = 800 nm. The contrast ratio between the main pulse and the nanosecond prepulse caused by the amplified spontaneous emission (ASE) was set to approximately 10^−9^. The picosecond contrast was on the order of 10^−4^–10^−5^.

### Experiment

The experimental setup is schematically shown in Fig. 1. The first laser was focused on the front edge of the slit nozzle of a He gas jet by a gold-coated off-axis parabolic (OAP) mirror with f/3.5 (*f* = 163 mm). The length of the gas jet was 1.2 mm. The stagnation pressure of the gas jet was set to 0.5–4 MPa, with the gas density at the laser axis on the order of *N* = 10^18^–10^19^ cm^−3^. The spatial distribution of the ejected electron beams was controlled by a phosphor screen (Mitsubishi Chemical Co. LTD, DRZ-High). The DRZ-High screen is sensitive to high-energy particles and radiation; thus, the front side of the screen was laminated with a 12 *μ*m thick aluminum foil to avoid exposure to the laser pulses, scattering lights and low-energy electrons. The scintillating images on these phosphor screens made by the deposited electrons were recorded by charged coupled device (CCD) cameras (Bitran Co., BU-51LN) with a commercial photographic lens from the backside of the screen.

To make a well-determined beam from the plasma cathode, we used a pulse solenoid lens system to extract electrons with low energy bands, similar to ref. ^25^. The parameters of the solenoid lens were chosen to provide the necessary focus ability for electron beams with an energy from 10 MeV. The solenoid was placed 100 mm after the injector plasma to collect and focus the electrons with the desired energy onto the booster plasma wake field 1 meter away.

The booster laser beam 2 was focused by an f/20 OAP mirror to another 1.2 mm gas jet with an intensity of 5 × 10^18^ W/cm^2^. A mirror with a 5 mm diameter hole at the center was installed in the electron beam line to reflect the booster laser beam while allowing the electrons from the cathode to pass through, as shown in Fig. 1. The temporal synchronization between these two laser beams was adjusted with the optical interference method, with a jitter measured to be less than 20 fs. An electron spectrometer (ESM) was installed just after the second gas jet.

### Particle-in-cell simulation

To explore the interaction of bunch electrons with the booster, we used numerical simulations with the 3D particle-in-cell code FPlaser3D^31^ to exploit the moving window technique. The postprocessing electrons in the bunches were separated from the plasma electrons to make the beam dynamics clearer and more understandable. For this purpose, the ‘beam’ electrons were specially marked. This allowed us to explore self-consistently and directly the beam dynamics and its relaxation. The calculations were performed with a spatial resolution of 2.5 × 10^−2^ *μ*m in the direction of laser pulse propagation and 7.5 × 10^−2^ μm in the transverse directions, and the temporal resolution was *Δt* = *Δx*/2. We used 25 particles per cell; the size of the simulation box was chosen to be large in both the transverse and longitudinal directions: 200 × 200 × 150 *μ*m^3^. This allowed us to explore the dynamics of practical, large beams propagating over cm length scale plasma.
